# Complete Genome Sequence of the Prototrophic Bacillus subtilis subsp. *subtilis* Strain SP1

**DOI:** 10.1128/MRA.00825-20

**Published:** 2020-08-06

**Authors:** Björn Richts, Robert Hertel, Sébastien Potot, Anja Poehlein, Rolf Daniel, Ghislain Schyns, Zoltán Prágai, Fabian M. Commichau

**Affiliations:** a Georg August University of Göttingen, Institute of Microbiology and Genetics, Department of General Microbiology, Göttingen, Germany; b Brandenburg University of Technology, FG Synthetic Microbiology, Cottbus-Senftenberg, Germany; c DSM Nutritional Products Ltd., Basel, Switzerland; d DSM Nutritional Products Ltd, Lexington, Massachusetts, USA; e Georg August University of Göttingen, Institute of Microbiology and Genetics, Department of Genomic and Applied Microbiology, Göttingen, Germany; f Georg August University of Göttingen, Institute of Microbiology and Genetics, Göttingen Genomics Laboratory, Göttingen, Germany; Broad Institute

## Abstract

Here, we present the complete genome sequence of the Bacillus subtilis strain SP1. This strain is a descendant of the laboratory strain 168. The strain is suitable for biotechnological applications because the prototrophy for tryptophan has been restored. Due to laboratory cultivation, the strain has acquired 24 additional sequence variations.

## ANNOUNCEMENT

The Bacillus subtilis strain SP1 is a descendant of the highly transformable laboratory strain 168. After the creation of B. subtilis strain 168 by X-irradiation, the strain developed genetic competence under laboratory conditions (1) and through this became a model bacterium for basic and applied research (2, 3). The plasmid-free strain was also subjected to genome reduction in order to identify the genes required for growth under defined conditions (4–6). However, the X-ray treatment made strain 168 auxotrophic for tryptophan due to a 3-bp deletion in the *trpC* gene (7, 8). The *trpC* gene encodes indole-3-glycerol phosphate synthase, which catalyzes an essential step in the *de novo* synthesis of tryptophan. The tryptophan prototrophy in the B. subtilis strain SP1 was restored by transforming strain 168 with a DNA fragment containing the wild-type *trpC* gene of the B. subtilis Marburg strain ATCC 6051 (9). The simplified medium requirements make strain SP1 suitable for industrial applications like vitamin production (9, 10) and basic research like examining the influence of substances that inhibit the biosynthesis of the aromatic amino acids phenylalanine, tryptophan, and tyrosine (11). To uncover all sequence variations between strain 168 and strain SP1 that might have occurred during the construction of SP1 (9), we have sequenced its genome.

The chromosomal DNA was isolated from growing cells using a commercially available kit (peqGOLD bacterial DNA kit; VWR International GmbH). Illumina (San Diego, CA, USA) paired-end sequencing libraries were generated with the Nextera XT DNA sample preparation kit and sequenced with the MiSeq system and reagent kit v.3 (2 × 300 bp) as recommended by the manufacturer (Illumina). Base calling was performed with MiSeq Control Software v.2.6.2.1. Default parameters were used for all software unless otherwise specified. The paired-end reads obtained (2.4 million) were quality processed and adapter trimmed with Trimmomatic v.0.39 (12). The 2.3 million high-quality paired-end reads recovered were used for single-nucleotide polymorphism analysis with Geneious Prime v.2020.0.5 (Biomatters, Ltd., Auckland, New Zealand) employing the genome of strain 168 (GenBank accession number NC_000964), as described (13, 14). Sequence deviations, identified with an average coverage of 135-fold and a minimum variant frequency of 0.98, confirmed the recovery of the *trpC* locus in strain SP1, which is responsible for its prototrophic phenotype, and revealed 24 additional genome modifications, which are summarized in Table 1. The identified sequence deviations were applied to the genome sequence of strain 168, which resembles the genome of strain SP1. The final genome sequence of SP1 was extracted with Geneious Prime v.2020.0.5 (Biomatters, Ltd.) and cross-verified with breseq v.0.35.1 (15) to ensure genome consistency and to confirm the absence of structural rearrangements. The single circular genome of SP1 resembled the genome of the ancestor strain 168 in all genetic properties except for the genome size, which was 7 bp larger for SP1, with 4,215,613 bp. Automated gene annotation was carried out by the Prokaryotic Genome Annotation Pipeline (PGAP) (16).

**TABLE 1:** Sequence variations identified in B. subtilis SP1 with respect to strain 168

Position^*a*^	Mutation	Locus^*b*^	Annotation
165748V165749	Δ1 bp	*HWV68_00950 → / → HWV68_00955*	Intergenic
165749V165750	Δ1 bp	*HWV68_00950 → / → HWV68_00955*	Intergenic
165824	+C	*HWV68_00955 →*	tRNA-Asn
166037	+T	*HWV68_00965 → / → HWV68_00970*	Intergenic
166343V166344	Δ1 bp	*HWV68_00980 → / → HWV68_00985*	Intergenic
557864	(T)5→6	*HWV68_02905 → / → HWV68_02910*	Intergenic
608214	(A)5→6	*HWV68_03190 ← / ← HWV68_03195*	Intergenic
1317153…1317154	+GT	*HWV68_06830 → / ← HWV68_06835*	Intergenic
1317156	(T)5→6	*HWV68_06830 → / ← HWV68_06835*	Intergenic
2097084	(A)7→8	*HWV68_10440 ← / ← HWV68_10445*	Intergenic
2271428	T→C	*HWV68_11655 ←*	UV damage repair protein (R78R [AGA→AGG])
2271509	C→T	*HWV68_11655 ←*	UV damage repair protein (V51V [GTG→GTA])
2271527	A→C	*HWV68_11655 ←*	UV damage repair protein (D45E [GAT→GAG])
2374563…2374565	+AAT	*HWV68_12285 ←*	Indole-3-glycerol phosphate synthase (*trpC*)
2480653…2480654	2 bp→AT	*HWV68_12900 → / ← HWV68_12905*	Intergenic
2480660V2480661	Δ1 bp	*HWV68_12900 → / ← HWV68_12905*	Intergenic
2480672V2480673	Δ1 bp	*HWV68_12900 → / ← HWV68_12905*	Intergenic
2581732	(T)6→7	*HWV68_13485 ← / ← HWV68_13490*	Intergenic
3010788	G→A	*HWV68_15835 ←*	SDR family oxidoreductase (T216I [ACA→ATA])
3391682	A→G	*HWV68_17820 →*	Spore germination receptor protein GerAA (T299A [ACA→GCA])
3391691	T→C	*HWV68_17820 →*	Spore germination receptor protein GerAA (S302P [TCC→CCC])
3770065	(A)8→9	*HWV68_19710 ← / ← HWV68_19715*	Intergenic
3935829…3935830	Δ1 bp	*HWV68_20570 → / ← HWV68_20575*	Intergenic
4095817	C→T	*HWV68_21345 ←*	GNAT family *N*-acetyltransferase (V9I [GTA→ATA])
4155397	(A)5→6	*HWV68_21665 ← / ← HWV68_21670*	Intergenic

a Coordinates refer to the position in the genome sequence of B. subtilis SP1 (GenBank accession number CP058242.1). V indicates a deleted base between the coordinates of the SP1 genome. Dots connecting coordinates indicate a range of inserted bases. Point mutations and single insertions are indicated with specific coordinates.

b The arrows indicate the gene directions.

### Data availability.

The genome sequence of the B. subtilis subsp*. subtilis* strain SP1 has been deposited in GenBank under the accession number CP058242.1. The raw sequence reads have been submitted to the NCBI Sequence Read Archive (SRA) database (17) under the accession number SRR12076664. The BioProject accession number is PRJNA641411, and the BioSample accession number is SAMN15352467.
